# The Mediating Role of Emotion Regulation Within the Relationship Between Neuroticism and Misophonia: A Preliminary Investigation

**DOI:** 10.3389/fpsyt.2020.00847

**Published:** 2020-08-28

**Authors:** Clair Cassiello-Robbins, Deepika Anand, Kibby McMahon, Rachel Guetta, Jacqueline Trumbull, Lisalynn Kelley, M. Zachary Rosenthal

**Affiliations:** ^1^Center for Misophonia and Emotion Regulation, Department of Psychiatry and Behavioral Sciences, Duke University Medical Center, Durham, NC, United States; ^2^Department of Psychology and Neuroscience, Duke University, Durham, NC, United States

**Keywords:** misophonia, emotion regulation, impulse control, impulsivity, neuroticism

## Abstract

Misophonia is a newly described condition characterized by heightened emotional reactivity (e.g., anger, anxiety, and disgust) to common repetitive sounds (e.g., oral or nasal sounds made by others), accompanied by difficulties responding to these sounds (e.g., intolerance, avoidance, and escape) and associated impairment in functioning. Although research indicates that problematic emotional responses are a key characteristic of misophonia, it is unknown whether individual differences in experiencing and regulating emotional responses influence severity of misophonia symptoms. Examination of individual differences in emotional functioning will help to guide treatment development for misophonia. Accordingly, the present study examined the associations among trait neuroticism, difficulties with emotion regulation, and symptoms of misophonia. For this study, a sample of 49 adults completed the Difficulties with Emotion Regulation Scale, the Misophonia Questionnaire, and the neuroticism subscale of the NEO–Personality inventory. Findings indicated that difficulties with emotion regulation and neuroticism were significantly positively correlated with symptoms of misophonia. Bootstrapped mediation analyses suggested that difficulties controlling impulsive behavior while experiencing intense negative emotions fully mediated the relationship between neuroticism and symptoms of misophonia. Results from this study suggest that neuroticism and difficulties with emotion regulation may be important risk factors and treatment targets for adults with misophonia, and difficulties controlling impulsive behavior when distressed may be an important individual difference accounting for the relationship between neuroticism and misophonia.

## Introduction

Misophonia, a condition originally described by Jastreboff and Jastreboff (1), is characterized by defensive motivational system responses (e.g., escape behavior, increased sympathetic arousal, and negative affect) to specific repetitive sounds and associated stimuli (1). Individuals with misophonia symptoms most commonly describe reactivity to repetitive sounds produced by humans (e.g., chewing, sniffing, and pen clicking) or the environment (e.g., clocks ticking) (2). These symptoms are linked to impairments in daily functioning and may even lead to other mental health problems [for a review, see Brout et al. (2)]. However, there are currently no evidence-based treatments for this condition. An important step in developing such interventions is to identify mechanisms (i.e., changeable psychological processes) that contribute to the development and maintenance of these symptoms and thus may be potential targets of treatment (3). A growing literature suggests that the way people experience and respond to strong emotions may be a promising candidate mechanism (2).

The strong, negative emotional reactions to specific sounds in misophonia often include anger and anxiety (4, 5). This response suggests neuroticism, the trait-like tendency to experience frequent and intense negative emotions in response to stressors, may be relevant to understanding misophonia (6). Previous research shows that people who are extremely reactive to noise also tend to be high on neuroticism (7, 8) and that high levels of neuroticism are associated with more bothersome tinnitus (9), although one study reported a non-significant relationship between neuroticism and general sound sensitivity (10). With regard to misophonia specifically, Jager et al. (11) reported individuals with misophonia scored above average on a measure of neuroticism and Daniels et al. (12) indicated a significant positive relationship between neuroticism and misophonia, with a small effect size [*r* = .32; (11, 12)]. Taken together, this literature suggests neuroticism may be a potential vulnerability factor for misophonia, as individuals high in neuroticism might be at risk for developing misophonic reactions to certain sounds they find aversive.

Beyond the experience of negative emotions, the way in which these emotions are managed may also play an important role in this mechanistic pathway to misophonia. The experience of negative emotions in and of itself is not problematic; indeed, emotions serve an adaptive function and can motivate productive behavioral responses. For example, anger can alert someone to a potential threat and motivate protective behavior (13). Emotions are typically managed through a process known as emotion regulation, a set of automatic and consciously mediated strategies that influence which emotions are experienced, the intensity of the emotion, and ways in which the emotions are expressed (14). Gratz and Roemer’s (15) influential model describes six specific components of emotion regulation, assessed using the Difficulties with Emotion Regulation Scale (DERS): (1) awareness of emotions when upset, (2) ability to differentiate specific emotions when upset, (3) non-judgmental acceptance of the experience of unpleasant emotions, (4) the ability to engage in goal-directed behavior while experiencing when upset, (5) the capacity to inhibit impulsive urges when upset, and (6) the belief that one has the ability to successfully use specific strategies (e.g., problem solving) to manage unpleasant emotions (15). When individuals have difficulty effectively utilizing these strategies, emotions may no longer serve their adaptive functions and instead become associated with distress and impairment.

A robust literature suggests a relationship exists between neuroticism and emotion regulation such that higher levels of neuroticism are associated with greater engagement in ineffective emotion regulation strategies (16–18). Given the intensity of negative emotionality following sounds and related stimuli in the context of misophonia, as well as preliminary research suggesting misophonia is associated with elevated levels of neuroticism, the ability to regulate emotions is likely essential to successful coping and management of this condition. For example, dysregulated anger in response to distressing sounds could result in frequent arguments and interpersonal aggression leading to tension in meaningful relationships and thus making misophonia even more impairing. Results from imaging studies support the hypothesis that emotion regulation is relevant to misophonia and report exaggerated responses in brain areas including the anterior insula cortex and amygdala, both of which are implicated in emotional processing and regulation (19, 20). Evidence from clinical research suggests that misophonia symptoms co-occur with a wide range of psychiatric disorders characterized by emotion dysregulation, including personality (e.g., borderline, avoidant, and obsessive-compulsive), depressive, anxiety (e.g., panic, agoraphobia, generalized anxiety, and social anxiety), and obsessive-compulsive disorders (11, 21–23). Therefore, misophonia may be especially related to clinically relevant difficulties with emotion regulation, which also have been shown to be transdiagnostic across a range of problems (24). At present, there are no evidence-based interventions specifically for misophonia. Research elucidating which specific facet(s) of emotion regulation difficulties influences misophonia will help identify the types of interventions that may be most effective.

Taken together, neuroticism and difficulties with emotion regulation are individual differences variables that may both play important roles in the etiology and maintenance of misophonia. Even further, it is important to examine the nature of these relationships to more thoroughly understand how tendencies to react emotionally can contribute to misophonia. Previous research has shown that difficulties with emotion regulation (as measured by the DERS) may account for the relationship between neuroticism and psychopathology. For example, Paulus et al. (25) found that difficulties with emotion regulation mediated the relationship between neuroticism and depression (25). Further, examination of specific emotion regulation components revealed that engagement in goal-directed behaviors, use of specific strategies, and impulse control each mediated the relationship between neuroticism and depression. Similarly, Mohammadkhani et al. (26) reported emotion dysregulation mediated the relationship between neuroticism and anxiety symptoms (26). Demonstrating a mediating role of a candidate mechanism provides evidence that targeting this mechanism with interventions may lead to desired therapeutic change (3). These previous findings suggest that emotion regulation interventions may improve symptoms of psychopathology in people who tend to experience negative emotions. This mechanistic relationship may also apply to those who suffer from misophonia.

To date, no studies have begun to investigate the relationships among neuroticism, difficulties with emotion regulation, and misophonia. Exploring these relationships may provide insight into how tendencies to experience and regulate emotions may be vulnerability factors for the onset or maintenance of misophonia. Accordingly, in an effort to help guide clinical interventions and research by identifying candidate transdiagnostic targets for treatment, the primary aim of this preliminary study was to examine the associations among trait neuroticism, difficulties with emotion regulation, and misophonia. We also explored how specific difficulties with emotion regulation may mediate this relationship. It was hypothesized that (1) neuroticism, difficulties with emotion regulation, and misophonia symptoms would be positively correlated and (2) the relationship between neuroticism and misophonia symptoms would be mediated by difficulties with emotion regulation.

## Method

### Participants

Participants (N = 49) were included from a parent study examining the relationship between self-reported symptoms of sensory processing dysfunction and mental health problems in adults [see McMahon et al. (27) for more information]. For this parent study, participants were recruited from the local community as part of a larger program of research studying difficulties with emotion regulation. Participants were identified *via* local online postings (including university and health center websites), newspaper postings, and referrals from mental health providers. Exclusion criteria included age (under 18), current mania, and/or presence of a psychotic disorder. Apart from these criteria, participants were not required to meet specific diagnostic or demographic criteria, thus allowing for a heterogeneous, transdiagnostic sample.

Most participants identified as female (*n* = 42), White (*n* = 32), and non-Hispanic (*n* = 46). The average age of the sample was 27.02 years (*SD* = 8.75). Most participants indicated they were single, never married (*n* = 35), had completed some college (*n* = 18), and had a salary range of $0–$10,000 (*n* = 27).

### Measures

#### Misophonia Questionnaire (MQ)

The MQ (5) contains 17 items within three subscales: misophonia symptomatology, related emotions and behaviors, and severity of sound sensitivity. The first two subscales inquire how well a series of describe the individual in general and are rated from 0 (not at all true) to 4 (always true) and are summed for a total score of 0–68. The third subscale asks individuals to provide a single rating of their sound sensitivity from 0 (no sound sensitivities) to 15 (very severe sound sensitivities). The total score reflects a summation of ratings from the misophonia symptomatology and related emotions and behaviors subscales, with higher scores reflecting greater overall sensitivity and distress. Initial validation of the MQ demonstrated good internal consistency (*α* = .86–.89) and internal consistency was also high in this sample (*α* = .93).

#### Difficulties in Emotion Regulation Scale (DERS)

The DERS (15) contains 36 self-report items that assess six facets of emotion dysregulation: (1) nonacceptance of emotional responses, (2) difficulties engaging in goal directed behavior while upset, (3) difficulties controlling impulsive behaviors while upset, (4) lack of emotional awareness, (5) limited access to emotion regulation strategies, and (6) lack of emotional clarity. Items assess how often various statements apply to an individual in general and responses are captured on a Likert scale that ranges from 1 (almost never) to 5 (almost always), with higher scores indicating greater difficulty with emotion regulation. The DERS has high internal consistency (Cronbach’s *α* = .93), good test-retest reliability (*r* = .88, *p <*.01), and adequate construct and predictive validity (15). In the current sample, internal consistency was high for the DERS total score (*α* = .96) as well as all subscales [range *α* = .89 (lack of emotional awareness) –.94 (nonacceptance of emotional responses)].

#### NEO–Personality Inventory (NEO-PI-3)

The NEO-PI-3 (28) is a 240-item questionnaire that assesses the Big Five personality traits (neuroticism, extraversion, openness, agreeableness, and conscientiousness). Neuroticism refers to the propensity to experience negative emotion (example item: “I often feel that I am not as good as others”), and though all five subscales were collected in the parent study, this is the only subscale used in the present study. The NEO-PI-3 is an updated version of the NEO-PI-R (29), with items now categorized into 30 facet scales across the five domains. All item responses are on a Likert scale ranging from 1 (strongly disagree) to 5 (strongly agree), with higher scores indicating greater tendency toward negative emotion in general. Psychometric properties of the NEO-PI-3 have been cross-culturally validated, with strong internal consistency, reliability, and validity (30). Internal consistency was strong in the current sample (*α* = .93).

### Procedures

The present study was conducted in a single laboratory visit. During their visit, participants met with a master’s level diagnostic assessor who had been rated to adherence. Upon arrival at the laboratory, participants provided written informed consent using protocols approved by the Institutional Review Board of the Duke University Medical Center. Participants completed interviews that assess for lifetime psychiatric diagnoses (Structured Clinical Interview for DSM-IV-TR Axis I Disorders [SCID I; (31)], treatment history, and sensory processing dysfunction. They also completed a battery of self-report questionnaires regarding emotional functioning, sensory processing, and psychiatric symptoms. The 49 participants who completed the Misophonia Questionnaire [MQ; (5)] were included in the present study.

### Data Analytic Plan

Data analyses were conducted in SPSS version 26 (32). Descriptive statistics were calculated, including tests of normality, and data that were not normally distributed was log transformed. The MQ total score was used to examine bivariate correlations of misophonia symptoms with neuroticism, as indicated by the Neuroticism subscale of the NEO-PI-3 and emotional dysregulation, as indicated by the DERS total score. Given a significant correlation between misophonia and global difficulties with emotion regulation, bivariate correlations examined whether misophonia symptoms were related to subscale scores of the DERS. Next, mediation models were examined using PROCESS, an SPSS macro for path-analysis based modeling (33), to test whether subscales with significant zero order correlations with misophonia symptoms mediated the relationship between neuroticism and misophonia symptom severity. The mediation model examined was Neuroticism subscale score from the NEO-PI-3 (IV)→ DERS-subscale (Mediator) → Misophonia Symptoms (MQ; DV), with each subscale modeled separately. All possible indirect paths were tested in these models.

Additionally, nonparametric bootstrapping was used to test the significance of indirect effects, in which the effect is interpreted as significant if 95% bias-corrected confidence intervals (CIs) for the effect do not include zero (34, 35). To adjust for the small sample size, mediation analyses were based on 5,000 bootstrapped samples [as recommended by Hayes (36)] using bias-corrected 95% CIs.

## Results

**Table 1** shows descriptive information for the study sample. In the current sample, the most frequent current DSM-IV diagnoses were Generalized Anxiety Disorder (n = 16), followed by Major Depressive Disorder (n = 9), Post-traumatic Stress Disorder (n = 9), Panic Disorder (n = 9), and then by Social Phobia (n = 5). Among Axis II diagnoses, the most common diagnosis was for Borderline Personality Disorder (n = 6), followed by Obsessive Compulsive Personality Disorder (n = 5)*.

**Table 1:** Sample characteristics (N = 49).

Variable	*M (SD)*	*N* (%)
Age	27.02 (8.75)	
Female		42 (85.7)
Race		
White		32 (65.3)
African American		8 (16.3)
Asian		7 (14.3)
More than one racial group		2 (4.1)
Income Level		
$0–$10,000		27 (55.1)
$10,001–$65,000		17 (34.6)
> $65,001		5 (10.2)
Education Level		
HS graduate or less		4 (8.2)
Vocational or some college		19 (38.7)
College graduate		12 (24.5)
Graduate school (in progress or completed)		14 (28.5)
DERS total score	97.35 (28.71)	
DERS Non-Acceptance Subscale	16.31 (7.12)	
DERS Goals Subscale	18.53 (5.30)	
DERS Impulse Control Subscale	12.57 (5.11)	
DERS Awareness Subscale	15.20 (5.77)	
DERS Strategies Subscale	22.29 (8.10)	
DERS Clarity Subscale	12.45 (4.50)	
MQ total score	97.35 (28.71)	
MQ Impairment Subscale	5.21 (2.40)	
MQ Sound Subscale	13.37 (8.19)	
MQ Reaction Subscale	13.55 (8.80)	
NEO-PI-3 Neuroticism Subscale	117.56 (31.43)	

SD, Standard Deviation; HS, High School; DERS, The Difficulties in Emotion Regulation Scale; MQ, Misophonia Questionnaire; NEO-PI-3, NEO Personality Inventory.

On average, the sample obtained a total DERS score of 97.35, a score 0.34 SDs higher than that reported in a sample of patients with heterogeneous DSM-5 diagnoses (37). The mean score on the impairment subscale of the MQ was 5.21, suggesting that the sample reported mild sound sensitivity, on average. Overall, the sample obtained a total neuroticism subscale score of 117.56, which is approximately 1 SD below that reported in a sample of treatment seeking adults (38). The DERS subscale indicating non-acceptance of emotional response (NONACCEPT) deviated from a normal distribution [skewness of 0.21 (SE = 0.34) and kurtosis of −1.16 (SE = 0.67)], therefore, it was log transformed to reduce bias [skewness of −0.36 (SE = 0.34) and kurtosis of −1.05 (SE = 0.67)].

### Bivariate Associations

Pearson correlations indicated the MQ total score (comprised of the symptomatology and related emotions and behaviors subscales) correlated significantly with the Neuroticism subscale of the NEO-PI-3 [*r*(45) = 0.38, *p* = 0.01] and the DERS total score [*r*(49) = 0.40, *p* < 0.01]. Neuroticism was significantly correlated with DERS total score [*r*(45) = 0.70, *p* < 0.01]. As hypothesized, these results indicate neuroticism, difficulties with emotion regulation, and misophonia symptoms were significantly positively correlated with each other. With regard to specific emotion regulation difficulties, MQ total score was significantly correlated with impulse control difficulties [*r*(49) = 0.30, *p* <.001], difficulties engaging in goal directed behavior [*r*(49) = 0.32, *p* <.001], lack of emotional clarity [*r*(49) = 0.38, *p* <.001], and limited perceived access to emotion regulation strategies [*r*(49) = 0.36, *p* <.001; see **Table 2**].

**Table 2:** Bivariate correlations between Misophonia symptoms and subscales of the Difficulty in Emotion Regulation Scale (DERS).

Variable	1	2	3	4	5	6	7
1. Misophonia Symptoms							
2. Goals	0.32*						
3. Impulse	0.38**	0.52**					
4. Awareness	0.27	0.33*	0.32*				
5. Strategies	0.36*	0.77**	0.70**	0.43**			
6. Clarity	0.38**	0.54**	0.49**	0.61**	0.71**		
7. Nonacceptance	0.17	0.57**	0.40**	0.43**	0.74**	0.63**	
8. Neuroticism	0.38*	.75**	.36*	.45*	.67**	.62**	.55**

Goals, Difficulties engaging in goal directed behavior; Impulse, Impulse control difficulties; Aware, Lack of emotional awareness; Strategies, Limited access to emotion regulation strategies; Clarity, Lack of emotional clarity; Nonacceptance, Nonacceptance of emotional response. *indicates p < 0.05. **indicates p < 0.01.

### Mediational Analyses

**Figure 1:**
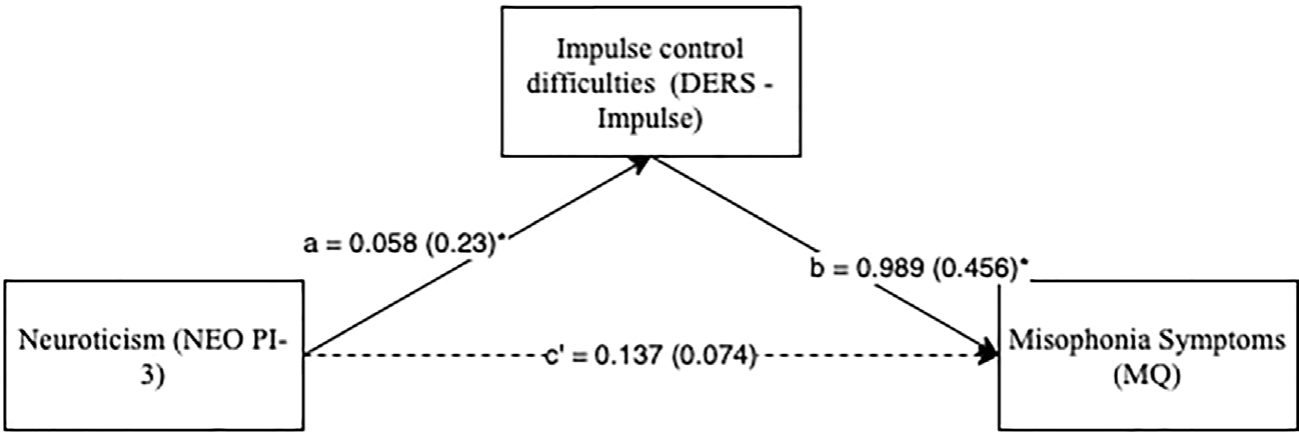
Neuroticism predicting misophonia symptoms with impulse control difficulties subscale as mediator. *p < 0.01. Standard errors are in parentheses; solid lines represent significant indirect paths; a = unstandardized regression coefficient for the IV predicting the mediator, b = unstandardized regression coefficient for the mediator predicting the DV with IV and mediator in the model, c’ = unstandardized coefficient for the IV predicting the DV with mediator in the model (direct effect); DERS -Impulse, Impulse Control Difficulties Subscale Score in Difficulty in Emotion Regulation Scale; MQ, Total Score in Misophonia Questionnaire; NEO PI-3, Neuroticism Subscale Score in NEO Personality Inventory-3.

Next, to identify whether any specific difficulties with emotion regulation best accounted for the relationship between neuroticism and misophonia symptoms, mediational analyses were conducted. As seen in **Figure 1**, high neuroticism significantly predicted difficulty controlling impulsive behavior when emotionally distressed, which, in turn, predicted higher misophonia symptoms. The indirect path (þ = 0.11; IE = 0.06, SE = 0.04, Bias Corrected 95% CI: LL = 0.01, UL = 0.15) from neuroticism to misophonia symptoms through the mediating effect of impulse control difficulties was significant [i.e., given that 0 was not included in the CI; (34, 35)]. Further, the direct path from neuroticism to misophonia symptoms (c = 0.20, SE = 0.07, t = 2.69, p < 0.05) was no longer significant (see **Figure 1**), suggesting that difficulties controlling impulses when experiencing strong unpleasant emotions fully mediated the relationship between neuroticism and misophonia.

The relationship between neuroticism and misophonia symptoms was not significantly mediated by DERS total score (β = 0.22, IE = 0.11, SE = 0.07, Bias Corrected 95% CI: LL = −0.02, UL = 0.26), non-acceptance of emotional responses (β = 0.05, IE = 0.03, SE = 0.05, Bias Corrected 95% CI: LL = −0.07, UL = 0.12), difficulties engaging in goal-directed behavior when upset (β = 0.07, IE = 0.04, SE = 0.08, Bias Corrected 95% CI: LL = −0.12, UL = 0.19), lack of emotional awareness (β = 0.07, IE = 0.03, SE = 0.04, Bias Corrected 95% CI: LL = −0.42, UL = 0.12), limited access to emotion regulation strategies when upset (β = 0.16, IE = 0.85, SE = 0.07, Bias Corrected 95% CI: LL = -0.04, UL = 0.22), or lack of emotional clarity (β = 0.17, IE = 0.09, SE = 0.05, Bias Corrected 95% CI: LL = −0.001, UL = 0.21).

## Discussion

The purpose of this study was to examine the relationships among neuroticism, difficulties with emotion regulation, and symptoms of misophonia. As predicted, misophonia symptoms were significantly and positively associated with neuroticism as well as difficulties with emotion regulation. In order to begin identifying specific candidate treatment targets for misophonia, we used Gratz and Roemer’s model of emotion regulation (15) to evaluate whether any specific components of emotion regulation could account for the relationship between higher neuroticism and misophonia symptoms. Results indicated that difficulties controlling impulsive behaviors when emotionally distressed fully mediated the relationship between neuroticism and misophonia symptoms.

Participants who reported higher symptoms of misophonia were more likely to endorse higher trait neuroticism, suggesting that those who tend to experience intense, frequent negative emotions more generally may be at higher risk for developing misophonia. These results extend previous research reporting a relationship between noise sensitivity and neuroticism (11, 12). In addition to this personality trait, self-reported difficulties regulating emotions were also significantly correlated with misophonia. Specifically, symptoms of misophonia had positive relationships with difficulties controlling impulsive behavior while experiencing intense emotions, difficulty engaging in goal-directed behavior, lack of access to emotion regulation strategies, and emotional clarity. These results are in line with research showing that this condition often co-occurs with disorders known to be associated with higher levels of neuroticism and emotion dysregulation, such as anxiety, depressive, and personality disorders (6). In our sample, the positive correlation between difficulties with emotion regulation and neuroticism was stronger than the relationship of either of these constructions with misophonia. These findings might suggest that the tendency to experience frequent, intense emotions and difficulties regulating them have a reciprocal relationship which confers risk for developing misophonia. The temporal relationship(s) between these constructs is of interest for future research studies.

Importantly, this is the first study to demonstrate that the relationship between trait neuroticism and misophonia is mediated by difficulty regulating impulsive behavior when emotionally distressed. People experiencing misophonia symptoms may respond to bothersome sounds and associated stimuli with impulsive aggressive behavior, such as yelling and indirect verbal aggression (23). A limited literature has examined impulse control in individuals with misophonia. This work suggests adults with misophonia favor accuracy over speed during cognitive tasks (e.g., stop-signal-task) under non-aversive conditions (39) but have poor cognitive control in response to misophonic sounds (12). Therefore, findings from this study further highlights inhibiting impulsive behavior when emotionally distressed as a candidate treatment target for individuals with misophonia symptoms. To further inform these intervention efforts, studies using multiple methodologies (e.g., behavioral, neuroscientific, and ecological momentary assessment) are needed to elucidate the role of biological and environmental contextual factors influencing specific difficulties regulating emotions (e.g., impulse control) when individuals with misophonia are emotionally distressed.

Results from the present study that highlight the role of emotion regulation in misophonia may have additional treatment implications. Evidence-based cognitive behavioral treatments, which intervene on difficulties with emotion regulation, may represent promising approaches to consider when treating misophonia. Indeed, most evidence-based psychological treatments include skills that facilitate awareness of one’s emotions, use of effective emotion regulation skills, impulse control, and engaging in goal-directed behavior, all of which were significantly correlated with higher misophonia symptoms in this study sample. Based on our findings, emotion regulation interventions that specifically target impulsive behaviors, such as the “STOP” or “Opposite action” skills from Dialectical Behavior Therapy (40), may be particularly useful for treating misophonia for individuals with high neuroticism. Learning and applying skills to regulate the experience of strong unpleasant emotions may help patients manage their distressing reactions to misophonic triggers in a goals-consistent way.

Results from this study should be considered in the context of their limitations. First, the sample size for this preliminary study was small, limiting the power of the analyses and generalizability of the findings. Although bootstrapping procedures were used to test mediational hypotheses, similar analyses with larger sample sizes are needed to replicate and extend the present findings. Second, this sample predominantly identified as female and White. Additional research should explore the relationships identified here in more diverse samples in order to understand how these relationships may or may not differ for individuals from different backgrounds. Third, the mediation analyses were conducted with cross-sectional data limiting our ability to draw conclusions about causal relationships between these variables, and the interaction among neuroticism, misophonia, and emotion regulation strategies over time. Indeed, the temporal relationship between neuroticism, emotion regulation, and misophonia is of interest to better understand the etiology and maintenance of misophonia. It is hypothesized that neuroticism and ineffective emotion regulation strategies confer vulnerability to developing misophonia and have a reciprocal relationship with one another. Rigorous experimental and longitudinal research is needed to shed light on this question. Research using longitudinal data would provide insight into the directional relationships among these variables. For example, research using daily repeated within-person measures over time (e.g., ecological momentary assessment) would indicate which specific emotion regulation strategies are effective or ineffective when individuals with misophonia symptoms are exposed to bothersome sounds and associated stimuli. Such discoveries would greatly inform treatment strategies for individuals with misophonia.

Despite limitations in this preliminary study, the results from this empirical study indicate relationships among neuroticism, difficulties with emotion regulation and misophonia. To our knowledge, this is the first study to demonstrate the mediating role of difficulties controlling impulsive behavior when emotionally distressed within the relationship between neuroticism and misophonia symptoms. We hope these results will help pave the way for future research to continue examining how individual differences (e.g., neuroticism) may be related to difficulties regulating emotions among individuals with misophonia. In particular, relationships among these variables need to be examined in large and diverse samples using prospective repeated measures and multiple methods of assessment. Such studies will, over time, provide answers to the current questions about how to provide treatments for individuals with misophonia.

## Data Availability Statement

The raw data supporting the conclusions of this article will be made available by the authors, without undue reservation.

## Ethics Statement

The studies involving human participants were reviewed and approved by Duke University Medical Center Institutional Review Board. The patients/participants provided their written informed consent to participate in this study.

## Author Contributions

All authors contributed to the article and approved the submitted version. DA conducted statistical analyses. CC-R and ZR revised and synthesized revisions of the manuscript from other authors.

## Funding

This study received funding from the Wallace Research Foundation.

## Conflict of Interest

ZR is a scientific advisor for BehaVR and Odin.

The remaining authors declare that the research was conducted in the absence of any commercial or financial relationships that could be construed as a potential conflict of interest.
